# Physical Intimacy in Older Couples’ Everyday Lives: Its Frequency and Links With Affect and Salivary Cortisol

**DOI:** 10.1093/geronb/gbac037

**Published:** 2022-03-14

**Authors:** Karolina Kolodziejczak, Johanna Drewelies, Theresa Pauly, Nilam Ram, Christiane Hoppmann, Denis Gerstorf

**Affiliations:** Department of Psychology, Humboldt University Berlin, Berlin, Germany; Department of Psychology, Humboldt University Berlin, Berlin, Germany; Department of Gender in Medicine, Charité Universitätsmedizin Berlin, Berlin, Germany; Department of Psychology, University of Zurich, Zürich, Switzerland; Departments of Psychology and Communication, Stanford University, Stanford, California, USA; Department of Psychology and Center for Hip Health & Mobility, University of British Columbia, Vancouver, British Columbia, Canada; Department of Psychology, Humboldt University Berlin, Berlin, Germany; The German Socio-Economic Panel (SOEP), German Institute for Economic Research (DIW), Berlin, Germany

**Keywords:** Affectionate touch, Physiological stress, Positive and negative emotions, Repeated within-person assessment, Romantic partners

## Abstract

**Objectives:**

Physical intimacy is important for communicating affection in romantic relationships. Theoretical and empirical work highlights linkages between physical intimacy, affect, and physiological stress among young and middle-aged adults, but not older adults. We examine physical intimacy and its associations with positive and negative affect and cortisol levels in the daily lives of older couples.

**Methods:**

We applied actor–partner multilevel models to repeated daily-life assessments of physical intimacy (experienced and wished) and affect obtained 6 times a day over 7 consecutive days from 120 older heterosexual German couples (*M*_age_ = 71.6, *SD*_age_ = 5.94). Physiological stress was indexed as total daily cortisol output, the area under the curve with respect to ground (AUC_g_).

**Results:**

Physical intimacy experienced and wished were reported at the vast majority of occasions, but to different degrees at different times. Within persons, in moments when participants experienced more physical intimacy, older women reported less negative affect, whereas older men reported more positive affect. Between persons, higher overall levels of physical intimacy experienced were associated with higher positive affect and less negative affect among women and with lower daily cortisol output among men. A stronger wish for intimacy was related to more negative affect among both women and men, and to higher daily cortisol output among men.

**Discussion:**

Physical intimacy is linked with mood and stress hormones in the daily life of older couples. We consider routes for future inquiry on physical intimacy among older adults.

Physical intimacy is intertwined with relational, psychological, and physical well-being, and thus constitutes an important component of close relationships (Burleson et al., 2013; Jakubiak & Feeney, 2017). Research with younger samples has shown that physical intimacy in everyday life, such as a hug or a kiss, is associated with elevated mood and reduced secretion of stress hormones (e.g., Ditzen et al., 2008), but we know little about the frequency and correlates of physical intimacy in the day-to-day lives of older adults. Importantly, the few studies available demonstrate that many older adults who have a partner often report experiencing physical intimacy (e.g., Freak-Poli, Kirkman, et al., 2017; Lee, Nazroo, et al., 2016). It is not yet known, though, how everyday physical intimacy in old age relates to time-varying indicators of well-being such as positive affect, negative affect, and stress hormone levels. To address these questions, we applied actor–partner multilevel models (Kenny et al., 2006) to daily-life data on intimacy, affect, and salivary cortisol from 120 heterosexual German couples aged 56–88 years (*M*_age_ = 71.6, *SD*_age_ = 5.94) obtained up to seven times per day over seven consecutive days.

## Everyday Physical Intimacy in Older Couples

Physical intimacy is linked with indicators of successful aging, such as social embeddedness (Kolodziejczak et al., 2019) or enjoyment of life (Smith et al., 2019), and thus requires more focus in aging research. Conceptual accounts suggest that one facet of physical intimacy is *affectionate touch*, defined as touch actually or typically demonstrating affection (e.g., love; care), for example, hugging, caressing, or kissing (Floyd, 2006; Jakubiak & Feeney, 2017). In some (operational) definitions, the nonsexual aspects of affectionate touch are emphasized (Galinsky et al., 2014; Gulledge et al., 2007); in other definitions affectionate touch is not aimed at immediate sexual gratification (Burleson et al., 2013), whereas elsewhere it is closely intertwined with sexuality (Smith et al., 2019). Following this literature, we refer to broadly defined everyday affectionate touch as *physical intimacy*.

Physical intimacy is an important channel for communicating affection throughout life, which helps maintain romantic relationships (Debrot et al., 2013; Gallace & Spence, 2010). Correspondingly, various forms of everyday intimacy are often reported by partnered older adults (Freak-Poli, Kirkman, et al., 2017; Lee, Nazroo, et al., 2016; Waite et al., 2009). However, these empirical studies have typically asked whether participants have experienced physical intimacy over the past 6–12 months. Such one-time retrospective reports cannot capture the interpersonal and intrapersonal dynamics that characterize how intimacy unfolds in older adults’ daily life. Additionally important is that the majority of older adults continue to desire intimacy in their 60s to 90s (Galinsky et al., 2014). For example, more than 80% of partnered German women and 90% of men in their mid-70s rated physical intimacy as important, and these numbers were higher than the ratings of sexual activity (Müller et al., 2014). This suggests that physical intimacy is highly valued and possibly desired by considerable proportions of older couples. Consequently, in the present study we examine both experienced and wished physical intimacy.

Importantly, physical intimacy takes place in a *dyadic* context. Accounting for factors from both partners helps capture the nature of daily-life partnered sexuality more accurately (Dewitte et al., 2015). Research from a dyadic perspective has demonstrated that, in older couples, the frequency of wishing for sexual activity was interrelated and correlated with the frequency of sexual activity (Waite et al., 2017). Likewise, both one’s own and the partner’s physical intimacy wished might be crucial for experiencing physical intimacy, which is in alignment with the idea that both partners would need to consent to intimacy. In turn, physical intimacy experienced might affect one’s well-being. For example, experiencing more physical intimacy was related to more positive affect (Debrot et al., 2013) and lower daily stress hormone levels in young and middle-aged couples (Ditzen et al., 2008). In older couples, partners’ physical intimacy wished might also be directly linked with indicators of well-being such as affect and (an absence of) physiological stress, similarly to how sexual desire is related to partnered older adults’ subjective well-being (see Lee, Vanhoutte, et al., 2016).

## Everyday Physical Intimacy and Affect in Older Couples

Conceptual perspectives have long posited that physical intimacy is closely intertwined with affect in romantic relationships (Gallace & Spence, 2010; Gulledge et al., 2007). Drawing from Jakubiak and Feeney’s (2017) work, we assume that physical intimacy contributes in many ways to well-being, both through neurobiological (e.g., via an upregulating hormone release, such as oxytocin and endogenous opioids) and relational-cognitive pathways (e.g., feeling valued and accepted). According to this, neuroendocrine and cognitive changes that occur in response to touch are expected to improve mood. Empirically, associations of everyday physical intimacy with affect have received less attention than those of sexual activity. However, the few studies available indicate that everyday intimacy is experienced more often than sexual activity among partnered individuals (e.g., Lee, Nazroo, et al., 2016) and is associated with positive affect and negative affect among young and middle-aged adults (Burleson et al., 2007; Ditzen et al., 2008). For older partnered individuals, initial evidence suggests that experiencing physical intimacy in the past 6 months is associated with higher positive affect (Freak-Poli, De Castro Lima, et al., 2017). Investigating the association between physical intimacy and affect in the daily life of older couples may help shed more light on relationship characteristics relevant for well-being in old age.

## Everyday Physical Intimacy and Physiological Stress in Older Couples

Another central notion of physical intimacy in romantic relationships is the buffering of stress. Again, conceptual accounts suggest that well-being and health benefits due to physical intimacy presumably occur through neurobiological and relational-cognitive pathways (Jakubiak & Feeney, 2017; Shrout, 2021). For example, the neuromodulator oxytocin that is released due to physical intimacy targets multiple areas in the brain and might induce, among others, feelings and cognitions of safety and belonging (Ditzen et al., 2019). This, in turn, downregulates physiological stress parameters. Correspondingly, in laboratory studies, gentle forms of intimacy between romantic partners (e.g., shoulder massage or hugging; holding hands during conflict discussions) have been found to lower people’s stress-induced cortisol levels, heart rate, and blood pressure (Ditzen et al., 2007, 2019; Gulledge et al., 2003; Light et al., 2005). In daily-life studies, *cortisol*, as a biomarker of stress that indexes activity of the hypothalamus–pituitary–adrenal axis (Piazza et al., 2010), is uniquely suited to highlight both between- and within-person characteristics that relate to stress reactivity (Hoppmann et al., 2018). Importantly, in daily-life studies, *salivary* cortisol assessments are relatively easy to implement and maximize ecological validity (Kudielka et al., 2012). Initial evidence exists that middle-aged couples who spent more time in physical intimacy exhibit lower daily salivary cortisol levels (Ditzen et al., 2008). This demonstrates the utility of salivary cortisol assessments in daily life for providing insights into the stress-buffering role of physical intimacy. To the best of our knowledge, links between physical intimacy and daily cortisol levels have not yet been examined among older couples.

## The Present Study

This study examines (1) how physical intimacy fluctuates in older couples’ daily lives and (2) how these fluctuations are associated with (a) self-reported positive affect and negative affect and (b) overall cortisol levels. To do so, we used data from 120 couples aged 56–88 years that reported momentary physical intimacy experienced and wished, and positive and negative affect. Additionally, participants provided salivary cortisol samples. Based on prior research on physical intimacy in the everyday lives of younger and middle-aged couples (Ditzen et al., 2008), we focused on overall daily cortisol secretion operationally defined by the area under the curve with respect to ground (AUC_g_: Pruessner et al., 2003). In our models, we controlled for variables known to influence daily emotions, cortisol profiles, and physical intimacy (including chronological age, education, body mass index [BMI], and relationship satisfaction: Gulledge et al., 2003; Hoppmann et al., 2018; Wrzus et al., 2013). We utilized gender as a distinguishing variable (Bolger & Laurenceau, 2013), but did not have any specific predictions regarding gender differences in the pattern of results. Drawing from previous literature that demonstrates how intimacy is linked with affect and cortisol levels among young and middle-aged adults (e.g., Burleson et al., 2007; Ditzen et al., 2008), we hypothesize that experiencing physical intimacy is associated with higher positive affect, lower negative affect, and lower salivary cortisol AUC_g_ levels in daily lives of older partnered adults. Additionally, we explore how both the actor’s and partner’s physical intimacy wished relates to changes in momentary affect and daily cortisol. Moreover, we test two-way interactions of physical intimacy, both experienced and wished, with other independent variables under study. We hypothesize that, for example, in moments when wish for intimacy is stronger than usual, experiencing more physical intimacy than usual correlates with more positive affect.

## Method

### Participants and Procedure

Participants consisted of 120 older heterosexual German couples recruited from the Socio-Economic Panel (Wagner et al., 2007). In 2018, trained interviewers contacted participants who fulfilled the eligibility criteria: Speaking German fluently; being around retirement age or older; living in a heterosexual relationship, married or cohabiting; having no vision or hearing impairments that could interfere with using an iPad; and having received treatment if participants had currently been diagnosed with hyper- or hypothyroidism. Studies with similar design and sample size (*n* = 87: Drewelies et al., 2020) showed significant actor and partner effects, suggesting that our study should provide sufficient statistical power to examine within-person associations (Bolger et al., 2011).

The protocol consisted of an introduction session, repeated daily-life assessments across seven consecutive days, and a Computer-Assisted Personal Interview. During a typical week, participants completed six short questionnaires per day (upon waking, at 10 a.m., 1 p.m., 4 p.m., 7 p.m., and 9 p.m.) using an iPad, and provided saliva samples seven times per day concurrent to the questionnaires and additionally 30 min after waking (so as to capture diurnal cortisol profiles: Nater et al., 2013). To avoid interference with daily routines, respondents were allowed to fill out questionnaires between 10 a.m. and 9 p.m. either 30 min prior or up to 120 min after the preset times (average deviation from scheduled times was 10 min, *SD* = 22.84). In the closing session, participants rated the study week as typical for their everyday lives (*M* = 4.08, *SD* = 1.02, ranging from 1 = “not at all” to 5 = “very typical”) and were compensated up to 100 Euros per person for completing all assessments. Further information on the study protocol can be found elsewhere (Pauly, Kolodziejczak, et al., 2021). Ethics approval for data collection was granted by the ethics committee of the Department of Psychology at Humboldt University Berlin.

### Measures

#### Physical intimacy

We assessed two aspects of physical intimacy. First, momentary *physical intimacy wished* with “Since the last questionnaire, how much did you wish to have some kind of physical intimacy (e.g., touching, hugging, or kissing) with your partner?”, answered using a 0 (“no particular wish”) to 100 (“strong wish”) sliding scale. Second, momentary *physical intimacy experienced* with “Since the last questionnaire, how much physical intimacy did you actually experience with your partner?”, rated on a 0 (“no intimacy at all”) to 100 (“much intimacy”) scale.

#### Positive and negative affect

Using the item “How (e.g., happy) do you feel right now?”, momentary *positive affect* was assessed with six items (mean across: “happy,” “interested,” “inspired,” “relaxed,” “balanced,” and “at rest”) and momentary *negative affect* with seven items (mean across: “depressed,” “disappointed,” “groggy,” “downcast”/“glum,” “overwhelmed,” “nervous,” and “jittery”), each answered using a 0 (“not at all”) to 100 (“strongly”) scale. The *select items* cover a broad range of low and high arousal emotions that have been shown in previous studies to: (a) fluctuate from one moment to the next, (b) be associated among older adults with other important daily-life constructs, such as perceived control or health sensitivity (e.g., Drewelies et al., 2020; Potter et al., 2021), and (c) exhibit good within-person reliabilities in our analysis sample (*R*_*C*_ = 0.74 for positive affect, *R*_*C*_ = 0.78 for negative affect; calculated as recommended by Cranford et al., 2006).

#### Salivary cortisol AUC_g_

Participants provided saliva samples using synthetic sticks in plastic tubes (Salivette^®^ Cortisol, Sarstedt, Nümbrecht, Germany), labeled to indicate day of study and time of assessment. Samples were stored during the study week in participants’ home freezer, afterwards at −31°C at Humboldt University Berlin, and subsequently shipped to Dresden LabService GmbH (Prof. Clemens Kirschbaum) for cortisol assaying; extremely low and high values were double-checked. The data were screened for compliance with the collection protocol (Hoppmann et al., 2018).

As an indicator of physiological stress, we calculated for each study day the area under the curve with respect to ground (AUC_g_), derived from the trapezoid formula using the discrete cortisol measurements and the time between measurements (Pruessner et al., 2003). We calculated AUC_g_ for days on which the two first cortisol measurements (upon waking and 30 min later) and in total, at least 3 cortisol measurements per day were available. Higher AUC_g_ scores can be interpreted as reflecting higher overall physiological stress levels (Hoppmann et al., 2018).

#### Covariates

*Age* was calculated as the difference between a participant’s year of birth and the year of data collection. *Education* was assessed as years of formal schooling. *BMI* was calculated as self-reported body weight in kilograms, divided by self-reported height in meters squared. *Relationship satisfaction* was assessed with the item: “All in all, how would you rate your current relationship?”, answered on a 5-point scale ranging from 1 (“very bad”) to 5 (“very good”). The utility of single-item measures of relationship satisfaction in large-scale studies has been shown elsewhere (Fülöp et al., 2020).

### Data Preparation

Participants provided valid data on both physical intimacy and affect on more than 9,780 occasions (e.g., physical intimacy experienced: *M* = 40.77 of 42 possible, *SD* = 2.30, range = 24–42). To model between-person differences and within-person fluctuations simultaneously, we separated the repeated assessments into time-invariant between-person variables (calculated as the person-specific mean over 42 occasions, i.e., *physical intimacy experienced* BP_*i*_ and *physical intimacy wished* BP_*i*_), and time-varying within-person variables (occasion-specific deviations from the person-specific mean for positive affect and negative affect as outcome variables, and day-specific deviations for salivary cortisol AUC_g_ as outcome variable, *physical intimacy experienced* WP_*ti*_ and *physical intimacy wished* WP_*ti*_; Bolger & Laurenceau, 2013). Acknowledging that intimacy takes place in a dyadic context (Hülür & Weber, 2019), we additionally created partner variables: *partner physical intimacy wished* BP_*i*_ and *partner physical intimacy wished* WP_*ti*_. Unconditional multilevel models revealed that 50% of the variance in momentary positive affect originated at the measurement occasion level, 32% at the between-person level, and 18% at the couple level. For momentary negative affect, the numbers were highly comparable (43%, 40%, and 17%, respectively).

Valid cortisol measurements were available on 11,405 occasions (*M* = 47.52 of 49 scheduled assessments, *SD* = 3.99, range = 14–49). As part of data cleaning, we winsorized cortisol (i.e., outliers of >±3 *SD* recoded as ±3 *SD*) and imputed missing values on occasions 3 through 7 (*n* = 145 occasions; 1.27%) using person-and-assessment-time-specific mean cortisol values (Wrosch et al., 2007). We replaced missing values on time intervals between assessments with the person-and-assessment-time-specific mean at occasions 1 and 2, and with the assessment-time-specific time interval at occasions 3 through 7 (180 or 120 min). For model convergence, we scaled the AUC_g_ cortisol variable at 1:100. The within-person predictors were centered at the person mean, age was centered at 70 years, and all other between-person predictors were centered at the sample mean. Unconditional models showed that 40% of the variance in the daily AUC_g_ originated at the day level, 42% at the person level, and 18% at the couple level.

### Data Analysis

We examined our research questions using repeated measures actor–partner interdependence models for distinguishable dyads, implemented in a multilevel modeling framework (Bolger & Laurenceau, 2013; Kenny et al., 2006). For the momentary positive affect outcome, we specified our models (subscript *w* for women; identical models for men and for negative affect) as:


$$\begin{array}{l} \begin{array}{ll} Positive\;affect_{tiw}= & \beta_{0iw}\\ & +\beta_{1iw}(physical\;intimacy\;experienced\;WP_{tiw})\\ & +\beta_{2iw}(physical\;intimacy\;wished\;WP_{tiw})\\ & +\beta_{3iw}(partner\;physical\;intimacy\;wished\;WP_{tiw})\\ & +e_{tiw}, \end{array} \end{array}$$ (1)

where positive affect reported at occasion *t* by woman *i* is a function of a person-specific intercept coefficient β _0*i*_ that indicates the expected value of the woman’s momentary positive affect; a person-specific slope coefficient β _1*i*_ that indicates the association between occasion-specific physical intimacy experienced and momentary positive affect; a person-specific slope β _2*i*_ that indicates the association between woman’s physical intimacy wished and positive affect; a person-specific slope β _3*i*_ that indicates the association between male partner’s physical intimacy wished and woman’s positive affect; and residual error, *e*_*ti*_. Between-person differences in the person-specific intercept coefficient β _0*i*_ were modeled as:


$$\begin{array}{l} \begin{array}{ll} \beta_{0iw}= & \gamma_{00w}+\gamma_{01w}(age_{iw})+\gamma_{02w}(education_{iw})\\ & +\;\gamma_{03w}(BMI_{iw})\\ & +\;\gamma_{04w}(relationship\;satisfaction_{iw})\\ & +\;\gamma_{05w}(physical\;intimacy\;experienced\;BP_{iw})\\ & +\;\gamma_{06w}(physical\;intimacy\;wished\;BP_{iw})\\ & +\;\gamma_{07w}(partner\;physical\;intimacy\;wished\;BP_{iw})\\ & +\;\gamma_{08w}(physical\;intimacy\;experienced\;BP_{iw}\\ & \times \;physical\;intimacy\;wished\;BP_{iw})+\;u_{0iw}, \end{array} \end{array}$$ (2)

and the person-specific coefficients β _1*i*_, β _2*i*_, and β _3*i*_ were modeled as:


$$\begin{array}{ll} \beta_{1iw}= & \;\gamma_{10w}+\gamma_{11w}(physical\;intimacy\;wished\;WP_{tiw})\\ & +u_{1iw}, \end{array}$$ (3)

$$\begin{array}{ll} \beta_{2iw}= & \;\gamma_{20w}+\gamma_{21w}(physical\;intimacy\;wished\;BP_{iw})\\ & +u_{2iw}, \end{array}$$ (4)

$$\begin{array}{l} \beta_{3iw}=\gamma_{30w}, \end{array}$$ (5)

where γ _00_ indicates the expected momentary positive affect scores for the prototypical older partnered woman in the sample; γ _10_ and γ _20_ represent prototypical within-person associations between woman’s momentary positive affect and physical intimacy experienced or physical intimacy wished, respectively; and γ _30_ indicates the prototypical association between the woman’s positive affect and her partner’s physical intimacy wished. Statistically significant two-way interactions, γ _08*w*_, γ _11*w*_, and γ _21*w*_ were identified in exploratory ways (for each outcome separately), and, in the final models, nonsignificant interactions (at alpha level of 0.05 for both women and men) were trimmed. The level-2 residuals, *u*_0*iw*_ and *u*_0*im*_, the level-1 residuals, *u*_1*iw*_ and *u*_1*im*_, and *u*_2*iw*_ and *u*_2*im*_, and the level-1 residual error terms, *e*_*tiw*_ and *e*_*tim*_, were allowed to covary,


$$\begin{array}{l} \left[\begin{array}{l} u_{0iw}\\ u_{0im} \end{array}\right]∼MVN\left(\begin{array}{ll} 0, & \left[\begin{array}{l} {\;\sigma }^{2}u_{0w}\\ \begin{array}{ll} \sigma u_{0w}u_{0m} & \;\sigma^{2}u_{0m} \end{array} \end{array}\right] \end{array}\right) \end{array}$$ (6)

$$\begin{array}{l} \left[\begin{array}{l} u_{1iw}\\ u_{1im} \end{array}\right]∼MVN\left(\begin{array}{ll} 0, & \left[\begin{array}{l} {\;\sigma }^{2}u_{1w}\\ \begin{array}{ll} \sigma u_{1w}u_{1m} & {\;\sigma }^{2}u_{1m} \end{array} \end{array}\right] \end{array}\right) \end{array}$$ (7)

$$\begin{array}{l} \left[\begin{array}{l} u_{2iw}\\ u_{2im} \end{array}\right]∼MVN\left(\begin{array}{ll} 0, & \left[\begin{array}{l} {\;\sigma }^{2}u_{2w}\\ \begin{array}{ll} \sigma u_{2w}u_{2m} & {\;\sigma }^{2}u_{2m} \end{array} \end{array}\right] \end{array}\right) \end{array}$$ (8)

$$\begin{array}{l} \left[\begin{array}{l} e_{tiw}\\ e_{tim} \end{array}\right]∼MVN\left(\begin{array}{ll} 0, & \left[\begin{array}{l} {\;\sigma }^{2}e_{w}\\ \begin{array}{ll} \sigma e_{w}e_{m} & {\;\sigma }^{2}e_{m} \end{array} \end{array}\right] \end{array}\right) \end{array}$$ (9)

Also, residuals were allowed to covary between successive occasions (autocorrelation). All equations described above were estimated simultaneously for women and men in a dyadic multilevel model.

For daily salivary cortisol AUC_g_, the within-person physical intimacy variables were configured as *day*-specific (instead of moment-specific) deviations from person-specific means (for details, see Supplementary Material). All models were estimated with SAS PROC MIXED (Littell et al., 2006) using restricted maximum likelihood estimation with missing data treated as missing at random (Little & Rubin, 1987).

## Results

Descriptive statistics and bivariate correlations for the variables under study are presented in Table 1. Participants (*N* = 240) were on average in their early 70s, predominantly married (97%), and in a long-term relationship (*M* = 46.5, *SD* = 11.2, range = 12–66 years). Relative to men, women reported on average lower positive affect (*d* = 0.44), physical intimacy experienced (*d* = 0.26), and physical intimacy wished (*d* = 0.60), and exhibited lower daily cortisol levels (*d* = 0.31). Experiencing more physical intimacy was associated with higher positive affect among both women (*r* = 0.34) and men (*r* = 0.37, both *p*s < 0.05) and with lower daily cortisol levels among men (*r* = −0.20 *p* < 0.05).

**Table 1.** Descriptive Statistics and Intercorrelations for the Variables Under Study

	Intercorrelations									
	(1)	(2)	(3)	(4)	(5)	(6)	(7)	(8)	(9)	(10)
(1) Salivary cortisol AUC_g_ (2.15–118.78)		−0.07	0.03	−0.04	0.08	−0.05	0.11	−0.20*	−0.04	−0.13
(2) Positive affect (32.05–96.56)	−0.06		−0.67*	0.18*	0.05	−0.05	0.39*	0.37*	0.16	0.20*
(3) Negative affect (0.08–65.53)	0.14	−0.62*		−0.09	−0.03	0.13	−0.41*	−0.13	0.14	−0.16
(4) Age (56–88)	0.13	−0.04	0.07		0.00	−0.23*	0.17	0.06	−0.02	0.05
(5) Education (1–17)	−0.05	0.12	−0.21*	−0.22*		−0.20*	0.09	−0.11	−0.03	0.03
(6) Body mass index (17.04–49.60)	−0.06	0.12	−0.09	−0.14	−0.03		−0.08	−0.02	−0.04	0.04
(7) Relationship satisfaction (1–5)	−0.07	0.30*	−0.35*	0.12	−0.01	0.01		0.33*	0.15	0.17
(8) Physical intimacy experienced (0–98.24)	−0.01	0.34*	−0.05	0.15	−0.14	−0.07	0.34*		0.73*	0.40*
(9) Physical intimacy wished (0.48–99.90)	−0.04	0.16	0.18*	0.10	−0.12	0.00	0.17	0.71*		0.38*
(10) Partner physical intimacy wished (0.48–99.90)	−0.02	0.11	0.07	−0.06	−0.10	0.07	0.08	0.45*	0.38*	
*M*_women_	47.83^a^	64.20^a^	17.46	70.28^a^	9.64^a^	26.60	4.29	33.64^a^	29.48^a^	44.01^a^
*SD*_women_	15.18	12.86	13.33	6.00	2.04	5.61	0.81	23.28	23.56	25.20
*M*_men_	53.05^a^	69.64^a^	16.45	72.83^a^	10.25^a^	27.37	4.38	39.73^a^	44.01^a^	29.48^a^
*SD*_men_	18.60	11.68	13.01	5.63	2.63	4.27	0.64	24.18	25.20	23.56

*Notes*: *N* = 120 couples (240 individuals) who provided on average data on approx. 41 occasions (on positive affect, negative affect, physical intimacy experienced, and physical intimacy wished) and approx. 48 saliva samples each. Intercorrelations for women presented below the diagonal, for men above the diagonal. All variables are between-person; values in brackets represent sample minimum and maximum. *M* = mean. *SD* = standard deviation. AUC_g_ = the area under the curve with respect to ground, scaled 1:100 for the purposes of the analysis. Positive affect = average of ratings for relaxed, balanced, at rest, happy, interested, inspired. Negative affect = average of ratings for depressed, disappointed, groggy, downcast/glum, overwhelmed, nervous, jittery. Age and education in years. Mean levels that differ between women and men (tested using ANOVA at *p* < .05 level) share the superscript (^a^).

*p < .05.

### Everyday Physical Intimacy in Older Couples

**Figure 1.**
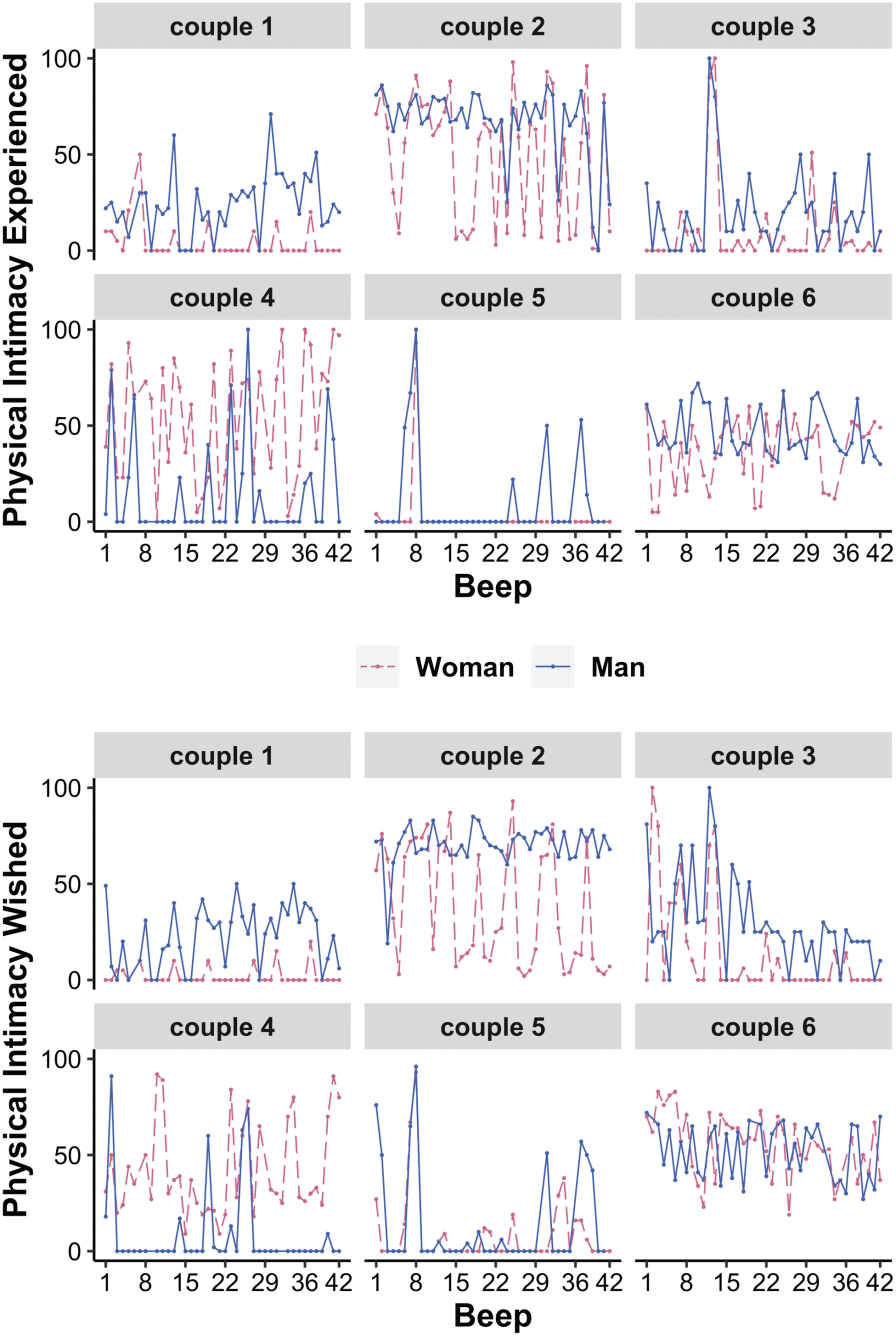
Distribution of responses on physical intimacy experienced (upper panel) and physical intimacy wished (bottom panel) over the course of the study. Data plotted for six randomly selected couples. It can be obtained that study participants differed in how much physical intimacy they experienced and wished for throughout the week and in how much their reports on intimacy fluctuated within days and across days. Full color version is available within the online issue.

Mean physical intimacy experienced across occasions was 36.61 (*SD* = 31.63, median = 30.00), mean physical intimacy wished was 36.63 (*SD* = 31.43, median = 31.00); both distributions were positively skewed (0.36–0.37). On 75% of all occasions, ratings of both experienced and wished physical intimacy were ≥ 6 (interquartile range = 59). Mean levels of both experienced and wished physical intimacy were highest between 9 p.m. and waking (*M* = 45.11; *SD* = 31.87 for physical intimacy experienced and *M* = 43.04; *SD* = 31.22 for physical intimacy wished) followed by the time between waking up and 10 a.m. (*M* = 40.07; *SD* = 31.92 and *M* = 38.47; *SD* = 31.38, respectively). The average within-person correlation between both intimacy variables was 0.46 (*SD* = 0.28). Example distribution of physical intimacy experienced and wished over the course of the study is depicted in Figure 1. It shows interindividual differences in how much physical intimacy fluctuated within and across days.

### Everyday Physical Intimacy and Affect in Older Couples

Results from actor–partner multilevel models for positive affect and negative affect as outcome variables are presented in Table 2. The prototypical level of momentary positive affect was γ _00*w*_ = 62.506 for women and γ _00*m*_ = 66.949 for men. As expected, among women, experiencing more physical intimacy was associated with more positive affect at the between-person level (γ _05*w*_ = 0.236), and among men, at the within-person level (γ _10*m*_ = 0.035). No significant associations between positive affect and physical intimacy wished (both actor and partner effects) were found. For the covariates, higher relationship satisfaction was associated with more positive affect among both women and men (γ _04*w*_ = 3.028, γ _04*m*_ = 3.857); no significant associations were found for age, education, and BMI. Additionally, several significant interaction effects occurred. For example, older women and men who reported on average more physical intimacy wished but less physical intimacy experienced also reported lower positive affect (γ _08*w*_ = 0.006, γ _08*m*_ = 0.004). Women’s and men’s intercepts were correlated 0.32, and the level-1 residuals 0.18. Fixed effects explained ≈ 22% of the variability in women’s and 24% variance in men’s positive affect.

**Table 2.** Multilevel Models Examining Positive Affect (Left-Hand), Negative Affect (Middle), and Total Daily Salivary Cortisol AUC_g_ (Right-Hand) Each as a Function of Physical Intimacy, Physical Intimacy Wished, Partner Physical Intimacy Wished, and Age, Education, Body Mass Index, and Relationship Satisfaction

	Positive affect				Negative affect				Salivary cortisol AUC_g_			
	Women		Men		Women		Men		Women		Men	
Parameter	Estimate	*SE*	Estimate	*SE*	Estimate	*SE*	Estimate	*SE*	Estimate	*SE*	Estimate	*SE*
Fixed effects												
Intercept, γ _00_	62.506*	1.382	66.949*	1.432	18.883*	1.412	17.170*	1.538	49.611*	1.861	49.339*	2.382
Age, γ _01_	−0.277	0.172	0.123	0.168	0.131	0.181	0.131	0.182	0.503*	0.252	−0.181	0.284
Education, γ _02_	0.433	0.491	0.309	0.358	−1.049*	0.521	0.136	0.390	0.260	0.699	−0.203	0.643
Body mass index, γ _03_	0.238	0.177	−0.112	0.220	−0.186	0.189	0.521*	0.240	−0.025	0.248	−0.179	0.365
Relationship satisfaction, γ _04_	3.028*	1.312	3.857*	1.568	−5.332*	1.367	−5.382*	1.705	−0.948	1.784	5.770*	2.595
Physical intimacy experienced BP, γ _05_	0.236*	0.066	0.080	0.068	−0.164*	0.070	−0.050	0.074	0.043	0.091	−0.392*	0.115
Physical intimacy experienced WP, γ _10_	0.022	0.014	0.035*	0.012	−0.027*	0.010	−0.027	0.014	−0.039	0.050	0.006	0.055
Physical intimacy wished BP, γ _06_	−0.080	0.061	−0.018	0.057	0.218*	0.014	0.190*	0.061	−0.035	0.085	0.257*	0.098
Physical intimacy wished WP, γ _20_	0.025	0.017	0.017	0.015	0.012	0.013	0.015	0.014	−0.054	0.054	−0.054	0.063
Partner physical intimacy wished BP, γ _07_	−0.046	0.047	0.006	0.045	0.032	0.047	−0.063	0.048	0.007	0.064	−0.125	0.075
Partner physical intimacy wished WP, γ _30_	−0.019	0.011	0.006	0.009	−0.002	0.010	0.011	0.008	0.049	0.050	−0.038	0.054
Physical intimacy experienced BP × Physical intimacy wished BP, γ _08_	0.006*	0.002	0.004*	0.002	−0.004*	0.002	−0.006*	0.002	−0.004*	0.002	0.007*	0.003
Age × Physical intimacy wished BP, γ _09_	—	—	—	—	—	—	—	—	0.009	0.011	−0.026*	0.011
Education × Physical intimacy wished BP, γ _010_	—	—	—	—	—	—	—	—	0.048	0.030	0.054*	0.027
Physical intimacy experienced WP × Physical intimacy wished WP, γ _11_	0.001*	0.001	0.001	0.001	—	—	—	—	—	—	—	—
Physical intimacy wished BP × Physical intimacy wished WP, γ _21a_	0.002*	0.001	0.003*	0.001	−0.002*	0.001	−0.003*	0.001	—	—	—	—
Age × Physical intimacy wished WP, γ _21b_	—	—	—	—	—	—	—	—	−0.016	0.008	0.019*	0.009
Random effects												
Between couples												
Variance intercept, σ ^2^_*u*0_	125.30*	17.899	101.26*	14.249	126.87*	17.935	115.29*	16.170	192.71*	31.365	247.15*	39.401
Variance physical intimacy experienced WP, σ ^2^_*u*1_	0.007*	0.003	0.005*	0.002	0.002	0.002	0.012*	0.003	—	—	—	—
Variance physical intimacy wished WP, σ ^2^_*u*2_	0.011*	0.004	0.010*	0.003	0.005*	0.002	0.010*	0.003	—	—	—	—
Covariance intercept women men, σ _*u*0*w*_,_*u*0*m*_	35.276*	12.037			20.183	12.875			81.664*	26.666		
Covariance physical intimacy experienced WP intercept, σ _*u*1_,_*u*0_	−0.151	0.164	−0.100	0.122	−0.067	0.129	−0.204	0.166	—	—	—	—
Covariance physical intimacy experienced WP women intercept men, σ _*u*1*w*_,_*u*0*m*_	−0.115	0.143			−0.093	0.115			—	—		
Covariance physical intimacy experienced WP men intercept women, σ _*u*1*m*_,_*u*0*w*_	0.070	0.132			−0.162	0.174			—	—		
Covariance physical intimacy experienced WP women men, σ _*u*1*w*_,_*u*1*m*_	0.001	0.002			−0.001	0.002			—	—		
Covariance physical intimacy wished WP intercept, σ _*u*2_,_*u*0_	−0.097	0.202	−0.158	0.153	−0.193	0.160	0.211	0.181	—	—	—	—
Covariance physical intimacy wished WP women intercept men, σ _*u*2*w*_,_*u*0*m*_	0.116	0.184			0.095	0.159			—	—		
Covariance physical intimacy wished WP physical intimacy WP, σ _*u*2_,_*u*1_	−0.003	0.003	−0.002	0.002	−0.001	0.001	−0.004*	0.002	—	—	—	—
Covariance physical intimacy wished WP women physical intimacy WP men, σ _*u*2*w*_,_*u*1*m*_	0.004	0.002			0.003	0.002			—	—		
Covariance physical intimacy wished WP men intercept women, σ _*u*2*m*_,_*u*0*w*_	−0.492*	0.171			−0.107	0.175			—	—		
Covariance physical intimacy wished WP men physical intimacy WP women, σ _*u*2*m*_,_*u*1*w*_	−0.001	0.003			0.001	0.002			—	—		
Covariance physical intimacy wished WP men physical intimacy wished WP women, σ _*u*2*m*_,_*u*2*w*_	−0.003	0.003			−0.003	0.002			—	—		
Within couples												
Residual variance, *e*_*ti*_	119.19*	2.577	182.02*	3.972	102.29*	2.290	144.49*	3.199	205.94*	12.056	185.55*	10.880
Residual covariance women men, *e*_*tiw, tim*_	37.584*	2.307			22.628*	1.863			35.239*	7.767		
Autocorrelation	0.176*	0.011			0.240*	0.011			0.186*	0.036		
Fit indices												
AIC	74,930.0				73,033.3				13,652.2			
−2LL	74,880.0				72,983.3				13,638.2			

*Notes*: *N* = 120 couples (240 individuals). Number of observations used in the momentary data model = 9,503. Number of observations used in the daily data model = 1,628. Estimate unstandardized. Positive affect = average of ratings for relaxed, balanced, at rest, happy, interested, inspired. Negative affect = average of ratings for depressed, disappointed, groggy, downcast/glum, overwhelmed, nervous, jittery. For model convergence, the salivary cortisol AUC_g_ variable was scaled at 1:100. −2LL = −2 res log likelihood; AIC = Akaike information criterion; AUC_g_ = the area under the curve with respect to ground; BP = between-person variable (person-specific mean over 42 occasions); *SE* = standard error; WP = within-person variable (occasion- or day-specific deviation from the person-specific mean). γ_21a_ = Interaction effect examined in model for positive/negative affect as outcome variable only. γ_21b_ = Interaction effect examined in model for as salivary cortisol AUC_g_ as outcome variable only.

*p < .05.

**Figure 2.**
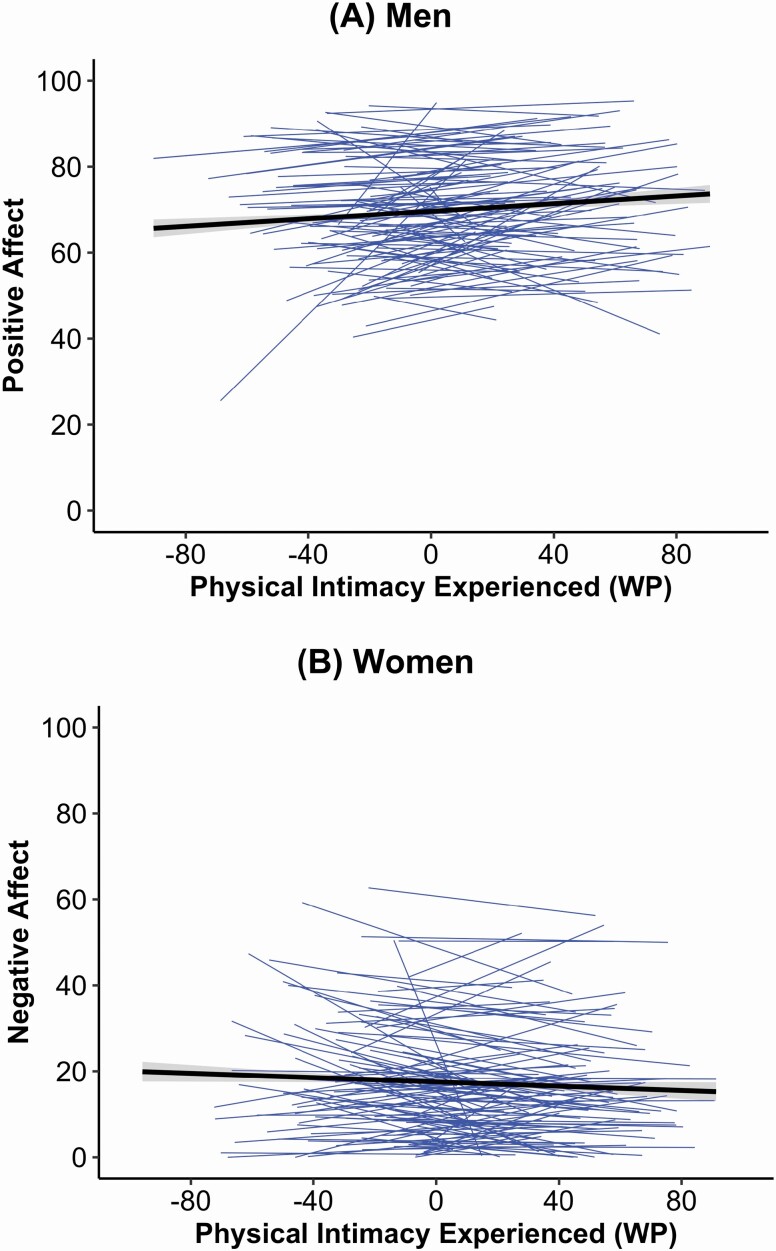
*N* = 120 couples (9,784 observations). Illustrating zero-order associations between physical intimacy experienced (within-person variable) and momentary positive affect among men (panel A) and momentary negative affect among women (panel B). It can be obtained that in moments of more physical intimacy than usual, men reported more positive affect and women reported less negative affect. Confidence intervals (95%) were represented around the regression line. WP = within-person variable. Full color version is available within the online issue.

The prototypical level of negative affect was γ _00*w*_ = 18.883 for women and γ _00*m*_ = 17.170 for men. Among women, experiencing more physical intimacy at both between-person and within-person level was associated with less negative affect (γ _05*w*_ = −0.164, γ _10*w*_ = −0.027). The within-person associations of physical intimacy experienced with negative affect among women are depicted in Figure 2, showing that in moments of experiencing more physical intimacy than usual, women reported less negative affect. Among men, no associations between physical intimacy experienced and negative affect were found. Women and men with higher overall levels of physical intimacy wished had higher negative affect (γ _06*w*_ = 0.218, γ _06*m*_ = 0.190). No partner effects emerged. For the covariates, higher education among women was related to lower negative affect (γ _02*w*_ = −1.049), and higher BMI among men was related to more negative affect (γ _03*m*_ = 0.521). Higher relationship satisfaction was associated with lower negative affect (γ _04*w*_ = −5.332, γ _04*m*_ = −5.382). Again, significant interactions occurred. For example, participants who reported on average more physical intimacy wished and on average less physical intimacy experienced, also reported more negative affect (γ _08*w*_ = −0.004, γ _08*m*_ = −0.006). Women’s and men’s intercepts were correlated 0.17, and the level-1 residuals 0.24. Fixed effects explained ≈ 27% of the variability in women’s and 30% in men’s negative affect.

### Everyday Physical Intimacy and Physiological Stress in Older Couples

**Figure 3.**
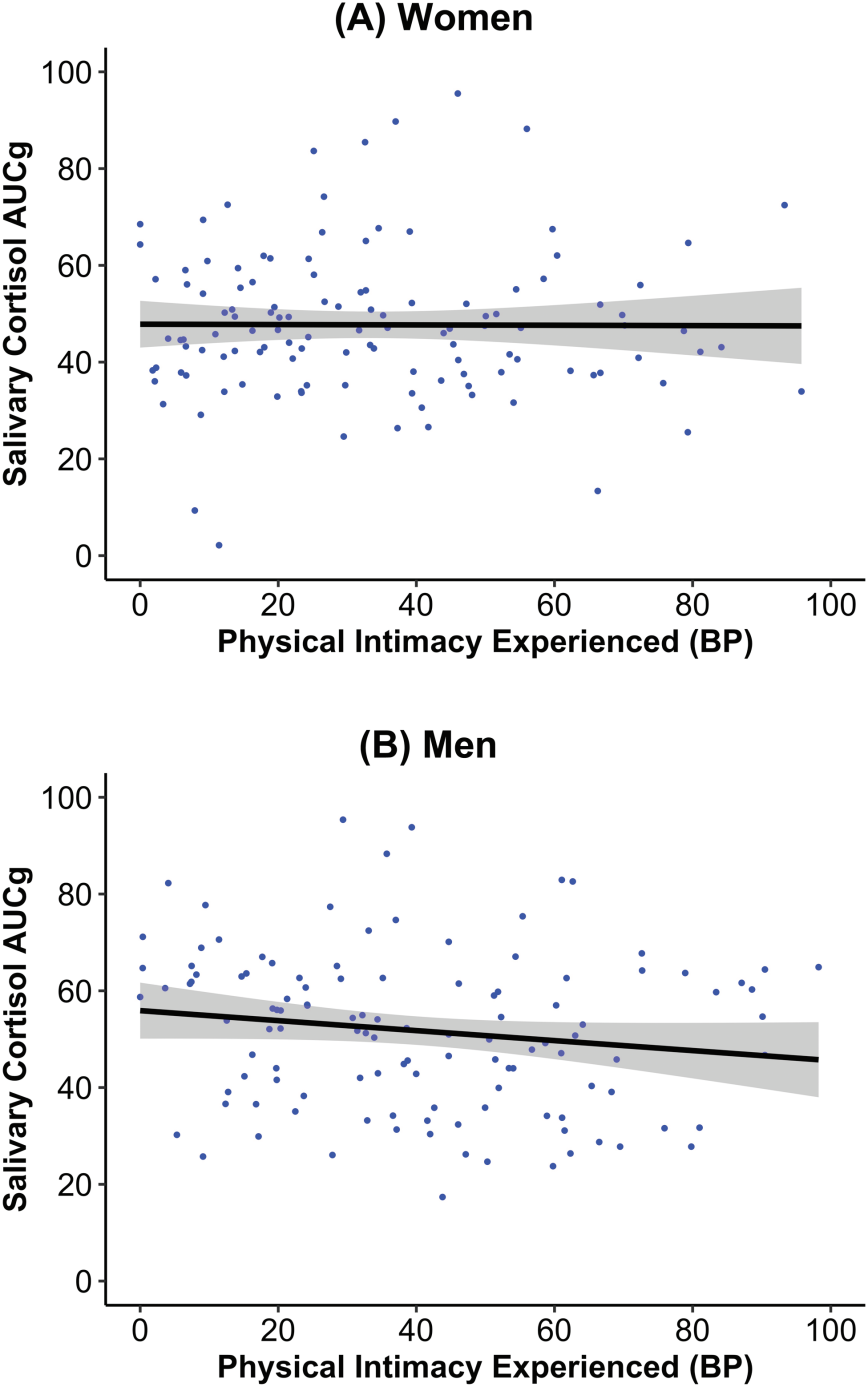
*N* = 120 couples (240 observations). Illustrating zero-order associations between mean physical intimacy experienced and the mean daily cortisol output calculated as the AUC_g_, separately for women (panel A) and men (panel B). It can be obtained that, at the between-person level, experiencing more physical intimacy was associated with lower daily cortisol levels among men, but not women. Confidence intervals (95%) were represented around the regression line. AUC_g_ = the area under the curve with respect to ground; BP = between-person variable. Full color version is available within the online issue.

Table 2 presents findings for cortisol. Prototypical daily cortisol output (scaled at 1:100) was γ _00*w*_ = 49.611 for women and γ _00*m*_ = 49.339 for men. As hypothesized, men with higher overall levels of physical intimacy had lower daily cortisol levels (γ _05*m*_ = −0.392), and men who reported more overall wish for intimacy also had higher cortisol levels (γ _06*m*_ = 0.257). Contrary to expectations, we found no significant associations of physical intimacy experienced with cortisol among women, and no within-person associations of physical intimacy experienced with cortisol among men. Considering the covariates, older age among women (γ _01*w*_ = 0.503) and higher relationship satisfaction among men (γ _04*m*_ = 5.770) were related to higher daily cortisol output. Between-person associations between physical intimacy experienced and cortisol AUC_g_ are depicted in Figure 3. For the interactions, for example, men who reported on average more physical intimacy wished and less physical intimacy experienced, also had higher daily cortisol outputs (γ _08*m*_ = 0.007). Women’s and men’s intercepts were correlated 0.37, and the level-1 residuals 0.19. Fixed effects explained ≈ 1% variance in women’s and ≈ 20% in men’s physiological stress levels.

## Discussion

Our objective was to provide further insights into the nature of physical intimacy and its associations with affect and physiological stress in the daily lives of older couples. Results revealed that in moments of more physical intimacy experienced, women reported less negative affect, and men reported more positive affect. For the between-person associations, women who experienced on average more physical intimacy reported more momentary positive affect and less negative affect. In turn, among men, more overall physical intimacy experienced was related to lower daily cortisol levels, and more overall physical intimacy wished was related to higher cortisol levels. In general, both women and men who reported on average more physical intimacy wished displayed more negative affect.

### Everyday Physical Intimacy in Older Couples

Mean levels of both experienced and wished physical intimacy dipped into the lower halves of the response scales. This suggests that, across all assessments, the levels of (experienced and wished) intimacy were relatively low, which is not surprising given the six assessments across any given day. At the same time, experiences of and wishes for intimacy were reported on the vast majority of occasions. Also, unsurprisingly, levels of experienced and wished intimacy differed by time of day, with more intimacy reported in the evenings and mornings (as shown earlier for sexual activity in adults aged 19–65 years; Dewitte et al., 2015). Considering that ≈ 90% (*n* = 109) of couples reported sharing a bedroom, the moments of physical proximity in bed may serve as a context that favors engaging in intimacy. On the other hand, exchanging physical intimacy might not be possible or desired when spending time on other activities during the day; for example, in public, or when one’s partner is not physically present. This might imply that physical intimacy remains an important channel for communicating affection in older romantic relationships; however, not all physical intimacy wished were enacted by participants, and the other way around, not all moments of experiencing physical intimacy were accompanied by intimacy wished. An avenue for future research should be to investigate correlates and implications of such discrepancies.

Importantly, there were both inter- and intraindividual differences in intimacy ratings. For example, the finding that men reported more physical intimacy experienced and wished than women presumably mirrors gender differences in the importance of physical intimacy (Müller et al., 2014) or the willingness to report intimacy. For the *within*-person differences, it is possible that physical intimacy occurs when partners exchange positive behaviors and interactions (Dewitte et al., 2015).

### Everyday Physical Intimacy and Affect in Older Couples

Consistent with findings from young and middle-aged adults (e.g., Burleson et al., 2007; Debrot et al., 2013; Ditzen et al., 2008), we found that more physical intimacy relates to more positive affect and less negative affect in the everyday lives of older couples. This implies that the previously identified linkages between physical intimacy and mood generalize to older adults. We note that reported gender differences in how physical intimacy experienced is associated with affect were identified in an exploratory manner, and should thus be interpreted with caution. Nevertheless, theoretical proposals have long argued for gender-specific linkages between intimacy and emotions in long-term relationships (Basson, 2000). For example, experiences of physical intimacy were less predictive for next-day positive mood among women than among men (Dewitte et al., 2015). With regard to the interaction effects, more physical intimacy wished and more physical intimacy experienced at momentary level were significantly associated with more positive affect only among women, and the effect was small in size. However, other interaction effects occurred among women and men, for example, the reported increased intimacy wished and less intimacy experienced at between-person level were associated with lower positive affect. Still, the significant interactions were identified in an exploratory fashion and were small in size; thus, they need to be corroborated in future research.

Interestingly, both women and men who on average reported more intimacy wished also reported more negative affect. This might reflect the discrepancy between intimacy desired and experienced for affect. Specifically, strong wish for bodily contact might result in negative affect when it does not go hand-in-hand with experiencing intimacy. However, because of the correlational nature of our analysis, it may also be that in moments of bad mood, the wish for being comforted by a hug from one’s partner increases. Although both items on intimacy were assessed on a 0%–100% scale, the labels of the scale endings differed. We thus decided not to create discrepancy measures between the two items, but rather opted to test for intimacy experienced and wished instead. It would be instructive for future research to examine whether greater discrepancy between intimacy experienced and desired predicts more negative affect. Also, future research might examine whether a greater discrepancy between actor’s and partner’s physical intimacy wished predicts actor’s negative affect.

Finally, to advance understanding of how the between- and within-day fluctuations in physical intimacy experienced and wished shape affect, we conducted follow-up analyses using *between-day* (person-and-day-specific mean over 6 occasions per day) and *within-day* (occasion-specific deviation from the person-and-day-specific mean) physical intimacy variables as predictors of positive affect and negative affect (see Supplementary Table S1). Results revealed that especially the between-day variable was a significant predictor of momentary affect. For example, on days where older adults experienced higher levels of physical intimacy, they also reported more positive affect and less negative affect. Informed by current results (of post-hoc analyses), we speculate that higher daily levels of physical intimacy, rather than within-day ups and downs, are related to elevated good mood of older partners.

### Everyday Physical Intimacy and Physiological Stress in Older Couples

Previous evidence on the linkages between physical intimacy and stress have primarily been obtained among women in experimental settings (e.g., Ditzen et al., 2019), or in young and middle-aged couples (e.g., Ditzen et al., 2008). To our knowledge, our study is the first to show that associations between everyday physical intimacy and physiological stress do not generalize to older women. Light and colleagues (2005) speculated that, among women, release of oxytocin and its beneficial effects for health and stress regulation might be substantially stronger prior to menopause than afterwards. In contrast, we found associations of experienced and wished physical intimacy with cortisol among older men. It is possible that experiencing physical intimacy buffers physiological stress in older men more strongly than in older women. For example, higher salivary cortisol levels were associated with increased psychological sexual arousal in young men (Goldey & van Anders, 2012). However, our study design does not allow for the investigation of the mechanisms underlying these gender differences.

Partner’s physical intimacy wished was not related to actor’s physiological stress. We speculate that this might be due to the subjectivity of people’s desires, which—if not communicated—would diminish the impact of physical intimacy wished on others’ mood and stress levels, as long as the wishes remain unexpressed. Previous studies suggest that open communication between older partners contributes to more satisfying sexual lives (Gillespie, 2017). Questions about how communicating intimate desires relates to well-being in everyday lives of older couples should be addressed in future research. Furthermore, it is possible that other partner variables are more central to one’s cortisol levels, such as partner’s cortisol levels (e.g., Saxbe & Repetti, 2010).

Prior research has reported that moments of physical and emotional closeness between partners are associated with greater cortisol synchrony (Pauly, Gerstorf, et al., 2021). Thus, future research could build on these findings by not only investigating whether cortisol levels are lower following moments of intimacy in daily life, but also whether cortisol and affect levels of both partners synchronize after such interactions. For modeling daily cortisol levels, the difference in women’s and men’s level-2 residuals’ correlation (estimated G correlation as produced by SAS PROC MIXED = 0.37) and level-1 residuals’ correlation (autocorrelation = 0.19) can be taken to indicate that constant effects (i.e., between-person) might play a more crucial role than temporary effects (i.e., within-person) for the shared variance in daily cortisol levels—if the numbers can indeed be compared directly across levels of analyses.

### Strengths, Limitations, and Outlook

The core strength of this project was the use of data from repeated assessments of older couples’ typical daily life, including assessments of physical intimacy and salivary cortisol. Another strength was the between-person and within-person levels of analysis, which enabled us to shed light on the underexplored topic of everyday physical intimacy and its correlates among older adults. However, our results do not allow us to draw temporal or causal inferences on how physical intimacy relates to affect and stress. For example, it is possible that in moments when people experience physical intimacy, their positive affect increases (similar to improved mood after sexual activity: Kashdan et al., 2018), but it is also possible that momentary good mood precedes engaging in intimate behavior, which additionally implies bidirectionality (Burleson et al., 2007; Dewitte et al., 2015). In our follow-up analyses, we proposed models that utilize positive affect, negative affect, and daily cortisol levels as predictor variables, and physical intimacy experienced and physical intimacy wished as outcome variables. We found some evidence that momentary affect, but not daily cortisol was related to experiencing and wishing for physical intimacy (for details, see Supplementary Tables S2 and S3). We note that employing lead-lag, time-ordered models would be required to examine whether physical intimacy experienced (or wished) at a given moment precedes more positive affect and less negative affect than usual at the next momentary measurement occasion. To approach a better understanding of possible bidirectionality, future research might also examine whether the size of the aforementioned effect is larger or smaller than the reverse direction of higher positive affect than usual at a given moment predicting physical intimacy experienced (or wished) at the next momentary measurement occasion.

Regarding the measures, our two single items captured peoples’ perceptions of physical intimacy, therefore providing more in-depth information on intimacy than frequency measures. In contrast, such measures do not allow clearly disentangling different types of behaviors (hug, kiss, etc.). Also, considering that both partners were asked about actual behaviors, we had expected these reports to overlap more strongly. Yet, the ratings sometimes differed between partners (see Figure 1), as did mean levels of reported intimacy. These discrepancies might highlight that “intimacy is in the eye of the beholder.” Though, it might be informative to consider such within-*couple* discrepancies more thoroughly. We speculate that larger everyday discrepancies in partners’ perceptions and needs for intimacy undermine relationship functioning (see Orr et al., 2019). Acknowledging that perceived stress and physiological markers of stress represent different and unique dimensions of the larger construct space (Campbell & Ehlert, 2012), we hypothesize that experiencing physical intimacy might be associated with *less* self-reported stress, and more physical intimacy wished with *more* self-reported stress (see also Jakubiak & Feeney, 2018). We also note that other stress dynamics may have emerged had we moved from cortisol as a physiological stress measure to cardiovascular outcomes (heart rate; blood pressure).

Finally, participants were in long-term, satisfying marital relationships. Thus, it is an open question whether our results generalize to less positively selected population segments. It would also be instructive to put our findings in perspective by examining physical intimacy among nonpartnered older adults. We speculate that for older singles other types of social relationships (e.g., emotional and instrumental support) are more important for well-being and quality of life (Thoits, 2011).

## Conclusion

This study used repeated assessment data obtained across seven consecutive days from couples aged 56–88 to examine time-varying associations of physical intimacy with positive affect, negative affect, and daily cortisol levels in the everyday lives of partnered older adults. As expected, results revealed that older partners wished for and experienced physical intimacy on a day-to-day basis, and that the extent of wishes and experiences fluctuated within and across days. Additionally, in moments of experiencing more physical intimacy than usual, older women and men reported less negative affect and older men experienced more positive affect. Higher mean levels of physical intimacy experienced were associated with more positive affect and less negative affect among older women, and lower daily cortisol levels among older men. Our findings extend previous research on intimacy in old age by applying a microlongitudinal perspective and contribute to the literature by demonstrating that physical intimacy is linked with positive and negative mood and stress hormone levels in the daily life of older couples. More mechanism-oriented research is needed to better understand the intricate links between everyday physical intimacy and well-being and gender differences therein among older adults.

## Supplementary Material

gbac037_suppl_Supplementary_Material
