# Supplementary material for: Physical Intimacy in Older Couples’ Everyday Lives: Its Frequency and Links With Affect and Salivary Cortisol
Source: J Gerontol B Psychol Sci Soc Sci. 2022 Mar 14;77(8):1416–30. doi: 10.1093/geronb/gbac037 (PMC9583184; doi:10.1093/geronb/gbac037)
Supplement: gbac037_suppl_Supplementary_Material [file gbac037_suppl_supplementary_material.pdf]

ONLINE SUPPLEMENT

Physical Intimacy in Older Couples' Everyday Lives: Its Frequency and Links with Affect and Salivary Cortisol

Karolina Kolodziejczak[1], Mag., Johanna Drewelies[1,2], PhD, Theresa Pauly[3], PhD

Nilam Ram[4], PhD, Christiane Hoppmann[5], PhD & Denis Gerstorf[1,6], PhD

[1]Humboldt University Berlin, Department of Psychology

[2]Charité Universitätsmedizin Berlin, Department of Gender in Medicine

[3]University of Zurich, Department of Psychology

[4]Stanford University, Departments of Psychology and Communication

[5]University of British Columbia, Department of Psychology and Center for Hip Health & Mobility

[6]German Institute for Economic Research (DIW)

Correspondence regarding this manuscript to: Karolina Kolodziejczak, Humboldt University Berlin, Department of Psychology, Unter den Linden 6, 10099 Berlin, Germany. E-mail: karolina.kolodziejczak@hu-berlin.de.

Data Analysis

For salivary cortisol as outcome variable, we specified our models (subscript *w* for women; identical models for men) as:

$$\text{Salivary cortisol AUC}_{gtiw} = \beta_{0iw} + \beta_{1iw}(\text{physical intimacy experienced WP}_{tiw}) + \beta_{2iw}(\text{physical intimacy wished WP}_{tiw}) + \beta_{3iw}(\text{partner physical intimacy wished WP}_{tiw}) + e_{tiw} \quad (1)$$

where salivary cortisol AUC_g reported at day *t* by woman *i* is a function of a person-specific intercept coefficient β_{0i} that indicates the expected value of woman's daily cortisol; a person-specific slope coefficient β_{1i} that represents the association between day-specific physical intimacy experienced and daily cortisol levels; a person-specific slope β_{2i} that indicates the association between woman's physical intimacy wished and daily cortisol levels; a person-specific slope β_{3i} that indicates the association between partner's physical intimacy wished and woman's daily cortisol levels; and residual error, e_{ti}. Between-person differences in the person-specific intercept coefficient β_{0i} were modeled as:

$$\beta_{0iw} = \gamma_{00w} + \gamma_{01w}(\text{age}_{iw}) + \gamma_{02w}(\text{education}_{iw}) + \gamma_{03w}(\text{BMI}_{iw}) + \gamma_{04w}(\text{relationship satisfaction}_{iw}) + \gamma_{05w}(\text{physical intimacy experienced BP}_{iw}) + \gamma_{06w}(\text{physical intimacy wished BP}_{iw}) + \gamma_{07w}(\text{partner physical intimacy wished BP}_{iw}) + \gamma_{08w}(\text{physical intimacy experienced BP}_{iw} \times \text{physical intimacy wished BP}_{iw}) + \gamma_{09w}(\text{age}_{iw} \times \text{physical intimacy wished BP}_{iw}) + \gamma_{010w}(\text{education}_{iw} \times \text{physical intimacy wished BP}_{iw}) + u_{0iw}, \quad (2)$$

and the person-specific coefficients β_{1i}, β_{2i}, and β_{3i} were modeled as:

$$\beta_{1iw} = \gamma_{10w}, \quad (3)$$

$$\beta_{2iw} = \gamma_{20w} + \gamma_{21w}(\text{age}_{iw}), \quad (4)$$

$$\beta_{3iw} = \gamma_{30w}, \quad (5)$$

where γ_{00} indicates expected daily cortisol levels for the prototypical older partnered woman (or man, respectively) in the sample; γ_{10} and γ_{20} represent prototypical within-person associations between woman's daily cortisol and physical intimacy experienced or wished, respectively, and γ_{30} indicates the prototypical within-couple association between woman's daily cortisol and her partner's physical intimacy wished. Final model includes statistically significant two-way interactions that were identified in an exploratory manner. Both the between-couple differences in level-2 residuals, u_{0iw} and u_{0im}, and the within-couple level-1 residual error terms, e_{tiw} and e_{tim}, were allowed to covary,

$$\begin{bmatrix} u_{0iw} \\ u_{0im} \end{bmatrix} \sim MVN\left(0, \begin{bmatrix} \sigma^2_{u0w} & \\ \sigma_{u0wu0m} & \sigma^2_{u0m} \end{bmatrix}\right) \quad (6)$$

$$\begin{bmatrix} e_{tiw} \\ e_{tim} \end{bmatrix} \sim MVN\left(0, \begin{bmatrix} \sigma^2_{e_w} & \\ \sigma_{e_w e_m} & \sigma^2_{e_m} \end{bmatrix}\right) \quad (7)$$

Also, residuals were allowed to covary between successive occasions (autocorrelation). All equations described above were estimated simultaneously for women and men in a dyadic multilevel model.

Table S1

Multilevel Models Examining Positive Affect (Left-Hand), and Negative Affect (Right-Hand) Each as a Function of Physical Intimacy Experienced (Between-Day and Within-Day), Physical Intimacy Wished (Between-Day and Within-Day), Partner Physical Intimacy Wished (Between-Day and Within-Day), and Age, Education, BMI, and Relationship Satisfaction

	Positive affect				Negative affect			
	Women		Men		Women		Men	
Parameter	Estimate	*SE*	Estimate	*SE*	Estimate	*SE*	Estimate	*SE*
Fixed effects								
Intercept, γ_{00}	65.105*	1.148	68.303*	1.128	17.406*	1.128	16.907*	1.288
Age, γ_{01}	–0.278	0.178	0.198	0.173	0.141	0.180	0.053	0.201
Education, γ_{02}	0.414	0.506	0.263	0.362	–1.036*	0.518	0.081	0.424
Body Mass Index, γ_{03}	0.227	0.182	–0.048	0.226	–0.179	0.187	0.433	0.266
Relationship satisfaction, γ_{04}	3.727*	1.293	4.998*	1.507	–5.276*	1.304	–7.371*	1.757
Physical intimacy experienced BD, γ_{05}	0.073*	0.022	0.069*	0.019	–0.067*	0.021	–0.051*	0.019
Physical intimacy experienced WD, γ_{10}	0.015	0.014	0.028*	0.012	–0.013	0.011	–0.021	0.011
Physical intimacy wished BD, γ_{06}	0.057*	0.023	0.054*	0.020	0.093*	0.022	0.007	0.020
Physical intimacy wished WD, γ_{20}	0.007	0.019	0.015	0.016	0.005	0.015	–0.008	0.015
Partner physical intimacy wished BD, γ_{07}	0.037	0.021	0.021	0.018	–0.050*	0.020	0.045*	0.018
Partner physical intimacy wished WD, γ_{30}	–0.032*	0.012	–0.001	0.010	0.009	0.011	0.003	0.009
Random effects								
Between couples								
Variance intercept, σ^2_{u0}	138.08*	19.405	108.24*	14.963	133.53*	18.725	142.46*	19.805
Variance physical intimacy experienced WD, σ^2_{u1}	0.007*	0.003	0.004*	0.002	0.002	0.002	0.004*	0.002
Variance physical intimacy wished WP, σ^2_{u2}	0.014*	0.005	0.012*	0.004	0.008*	0.003	0.010*	0.003
Covariance intercept women men, $\sigma_{u0w,\ u0m}$	41.701*	12.862			22.826	14.497		
Covariance physical intimacy experienced WD intercept, $\sigma_{u1,\ u0}$	–0.359	0.185	–0.117	0.128	–0.153	0.144	–0.207	0.152
Covariance physical intimacy experienced WD women intercept men, $\sigma_{u1w,\ u0m}$	–0.079	0.148			0.077	0.129		
Covariance physical intimacy experienced WD men intercept women, $\sigma_{u1m,\ u0w}$	–0.051	0.144			–0.083	0.143		

Covariance physical intimacy experienced WD women men, $\sigma_{u1w, u1m}$	0.001	0.002			–0.001	0.001		
Covariance physical intimacy wished WD intercept, $\sigma_{u2, u0}$	0.063	0.231	–0.166	0.172	–0.192	0.198	–0.095	0.205
Covariance physical intimacy wished WD women intercept men, $\sigma_{u2w, u0m}$	–0.036	0.197			–0.004	0.186		
Covariance physical intimacy wished WD physical intimacy experienced WD, $\sigma_{u2, u1}$	–0.001	0.003	–0.001	0.002	–0.001	0.002	–0.002	0.002
Covariance physical intimacy wished WD women physical intimacy experienced WD men, $\sigma_{u2w, u1m}$	0.006*	0.003			0.002	0.002		
Covariance physical intimacy wished WD men intercept women, $\sigma_{u2m, u0w}$	–0.419*	0.198			–0.371*	0.187		
Covariance physical intimacy wished WD men physical intimacy experienced WD women, $\sigma_{u2m, u1w}$	–0.005	0.003			0.001	0.002		
Covariance physical intimacy wished WD men physical intimacy wished WD women, $\sigma_{u2m, u2w}$	–0.001	0.004			0.001	0.003		
Within couples								
Residual variance, e_{ti}	120.16*	2.596	181.96*	3.983	106.62*	2.360	145.73*	3.238
Residual covariance women men, $e_{tiw, tim}$	37.578*	2.317			23.061*	1.904		
Autocorrelation	0.181*	0.011			0.258*	0.011		
Fit indices								
AIC	74,893.9				73,098.0			
–2LL	74,843.9				73,048.0			

Note. N = 120 couples (240 individuals). Number of observations used in the momentary data model = 9,503. Estimate unstandardized. Positive affect = average of ratings for relaxed, balanced, at rest, happy, interested, inspired. Negative affect = average of ratings for depressed, disappointed, groggy, downcast/glum, overwhelmed, nervous, jittery. For model convergence, the salivary cortisol AUC_g variable was scaled at 1:100. *SE* = Standard Error; BD = Between-day variable (person-and-day-specific mean over 6 occasions per day); WD = Within-day variable (occasion-specific deviation from the person-and-day-specific mean); AIC = Akaike information criterion; –2LL = –2 Res Log Likelihood.

$^*p < .05$

Table S2

Multilevel Models Examining Momentary Physical Intimacy Experienced as a Function of Positive Affect (Model 1), Negative Affect (Model 2), or Salivary Cortisol AUC_g (Model 3), and Age, Education, BMI, and Relationship Satisfaction

	Physical Intimacy Experienced											
	Model 1				Model 2				Model 3			
	Women		Men		Women		Men		Women		Men	
Parameter	Estimate	*SE*	Estimate	*SE*	Estimate	*SE*	Estimate	*SE*	Estimate	*SE*	Estimate	*SE*
Fixed effects												
Intercept, γ_{00}	2.927	9.391	6.034	12.035	32.742*	3.179	38.610*	3.503	33.179*	6.053	51.812*	5.901
Age, γ_{01}	0.336	0.309	0.028	0.353	0.291	0.320	0.214	0.353	0.320	0.329	0.048	0.356
Education, γ_{02}	–1.121	0.872	–1.220	0.725	–0.771	0.919	–1.011	0.727	–0.904	0.910	–0.996	0.732
Body Mass Index, γ_{03}	–0.539	0.311	–0.091	0.449	–0.448	0.321	0.220	0.453	–0.490	0.323	–0.196	0.451
Relationship satisfaction, γ_{04}	5.917*	2.286	6.439*	3.198	7.950*	2.434	9.155*	3.225	8.280*	2.329	9.822*	3.059
Positive affect BP, γ_{05}[a]	0.472*	0.142	0.488*	0.172	–	–	–	–	–	–	–	–
Positive affect WP, γ_{10}[a]	0.062*	0.028	0.123*	0.031	–	–	–	–	–	–	–	–
Negative affect BP, γ_{05}[b]	–	–	–	–	0.046	0.150	0.048	0.154	–	–	–	–
Negative affect WP, γ_{10}[b]	–	–	–	–	–0.047	0.030	–0.094*	0.035	–	–	–	–
Salivary cortisol AUC_g BP, γ_{05}[c]	–	–	–	–	–	–	–	–	0.012	0.123	–0.203	0.104
Salivary cortisol AUC_g WP, γ_{10}[c]	–	–	–	–	–	–	–	–	–0.004	0.029	–0.023	0.025
Random effects												
Between couples												
Variance intercept, σ^2_{u0}	420.94*	58.353	477.61*	66.122	467.07*	64.380	525.70*	72.735	461.62*	63.662	490.51*	68.458
Variance predictor WP[a,b,c], σ^2_{u1}	0.031*	0.113	0.035*	0.014	0.021	0.014	0.044*	0.016	–	–	–	–
Covariance intercept women men, $\sigma_{u0w,\ u0m}$	204.97*	50.274			254.97	55.979			232.93*	53.643		
Covariance predictor WP[a,b,c] intercept, $\sigma_{u1,\ u0}$	0.164	0.596	0.920	0.710	–0.167	0.687	–2.476*	0.891	–	–	–	–
Covariance predictor WP[a,b,c] women intercept men, $\sigma_{u1w,\ u0m}$	–0.614	0.620			0.246	0.688			–	–		
Covariance predictor WP[a,b,c] men intercept women, $\sigma_{u1m,\ u0w}$	1.605*	0.671			–1.912*	0.836			–	–		
Covariance predictor WP[a,b,c] women men, $\sigma_{u1w,\ u1m}$	0.006	0.010			0.007	0.011			–	–		

Within couples												
Residual variance, e_{ti}	407.81*	8.660	465.32*	9.969	407.95*	8.654	467.90*	10.017	418.72*	8.866	473.20*	10.118
Residual covariance women men, $e_{tiw, tim}$	144.04*	6.793			144.05*	10.017			146.11*	6.941		
Autocorrelation	0.204*	0.011			0.201*	0.011			0.204*	0.011		
Fit indices												
AIC	86,439.8				86,472.4				85,066.4			
–2LL	86,411.8				86,444.4				85,052.4			

Note. *N* = 120 couples (240 individuals). Number of observations used in the positive affect/negative affect models = 9,702. Number of observations used in the salivary cortisol AUC_g model = 9,532. Estimate unstandardized. Positive affect = average of ratings for relaxed, balanced, at rest, happy, interested, inspired. Negative affect = average of ratings for depressed, disappointed, groggy, downcast/glum, overwhelmed, nervous, jittery. For model convergence, the salivary cortisol AUC_g variable was scaled at 1:100. *SE* = Standard Error; BP = Between-person variable (person-specific mean over 42 occasions); WP = Within-person variable (occasion- or day-specific deviation from the person-specific mean); AIC = Akaike information criterion; –2LL = –2 Res Log Likelihood.

[a] positive affect as predictor variable of interest

[b] negative affect as predictor variable of interest

[c] salivary cortisol AUC_g as predictor variable of interest

*$p < .05$.

Table S3

Multilevel Models Examining Momentary Physical Intimacy Wished as a Function of Positive Affect (Model 1), Negative Affect (Model 2), or Salivary Cortisol AUC_g (Model 3), and Age, Education, BMI, and Relationship Satisfaction

	Physical Intimacy Wished											
	Model 1				Model 2				Model 3			
	Women		Men		Women		Men		Women		Men	
Parameter	Estimate	*SE*	Estimate	*SE*	Estimate	*SE*	Estimate	*SE*	Estimate	*SE*	Estimate	*SE*
Fixed effects												
Intercept, γ_{00}	13.653	10.799	30.184*	14.001	23.138*	3.363	35.218*	3.798	31.734*	6.806	47.639*	6.978
Age, γ_{01}	0.438	0.352	–0.151	0.407	0.333	0.340	–0.096	0.381	0.383	0.364	–0.222	0.415
Education, γ_{02}	–0.475	1.004	–0.694	0.842	–0.326	0.983	–0.790	0.791	–0.790	1.025	–0.622	0.865
Body Mass Index, γ_{03}	–0.168	0.360	–0.274	0.523	–0.113	0.344	–0.723	0.494	–0.067	0.367	–0.380	0.537
Relationship satisfaction, γ_{04}	3.668	2.603	2.239	3.705	6.376*	2.579	7.732*	3.501	4.764	2.579	5.613	3.594
Positive affect BP, γ_{05}[a]	0.242	0.163	0.212	0.201	–	–	–	–	–	–	–	–
Positive affect WP, γ_{10}[a]	0.064*	0.029	0.106*	0.033	–	–	–	–	–	–	–	–
Negative affect BP, γ_{05}[b]	–	–	–	–	0.367*	0.160	0.578*	0.168	–	–	–	–
Negative affect WP, γ_{10}[b]	–	–	–	–	0.016	0.032	–0.047	0.035	–	–	–	–
Salivary cortisol AUC_g BP, γ_{05}[c]	–	–	–	–	–	–	–	–	–0.010	0.141	–0.039	0.123
Salivary cortisol AUC_g WP, γ_{10}[c]	–	–	–	–	–	–	–	–	–0.042	0.027	–0.012	0.025
Random effects												
Between couples												
Variance intercept, σ^2_{u0}	526.20*	71.988	621.83*	84.641	508.44*	69.332	604.74*	82.715	534.61*	72.982	628.38*	85.454
Variance predictor WP[a,b,c], σ^2_{u1}	0.050*	0.013	0.048*	0.014	0.049*	0.015	0.054*	0.018	–	–	–	–
Covariance intercept women men, $\sigma_{u0w,\,u0m}$	208.08*	58.778			234.90*	58.408			214.18*	59.654		
Covariance predictor WP[a,b,c] intercept, $\sigma_{u1,\,u0}$	1.371	0.708	1.687	0.836	–1.211	0.771	–2.664*	0.953	–	–	–	–
Covariance predictor WP[a,b,c] women intercept men, $\sigma_{u1w,\,u0m}$	–0.346	0.740			–0.124	0.809			–	–		
Covariance predictor WP[a,b,c] men intercept women, $\sigma_{u1m,\,u0w}$	0.441	0.783			0.183	0.872			–	–		
Covariance predictor WP[a,b,c] women men, $\sigma_{u1w,\,u1m}$	–0.007	0.011			0.006	0.012			–	–		

Within couples												
Residual variance, e_{ti}	348.36*	7.528	345.72*	7.484	349.46*	7.555	348.93*	7.537	359.08*	7.746	357.35*	7.719
Residual covariance women men, $e_{tiw,\ tim}$	60.749*	5.201			61.544*	5.224			61.264*	5.360		
Autocorrelation	0.250*	0.011			0.247*	0.011			0.250*	0.011		
Fit indices												
AIC			84,565.4				84,597.3				83,254.9	
–2LL			84,537.4				84,569.3				83,240.9	

Note. N = 120 couples (240 individuals). Number of observations used in the positive affect/negative affect models = 9,702. Number of observations used in the salivary cortisol AUC_g model = 9,532. Estimate unstandardized. Positive affect = average of ratings for relaxed, balanced, at rest, happy, interested, inspired. Negative affect = average of ratings for depressed, disappointed, groggy, downcast/glum, overwhelmed, nervous, jittery. For model convergence, the salivary cortisol AUC_g variable was scaled at 1:100. *SE* = Standard Error; BP = Between-person variable (person-specific mean over 42 occasions); WP = Within-person variable (occasion- or day-specific deviation from the person-specific mean); AIC = Akaike information criterion; –2LL = –2 Res Log Likelihood.

[a] positive affect as predictor variable of interest

[b] negative affect as predictor variable of interest

[c] salivary cortisol AUC_g as predictor variable of interest

*p < .05.
